# Evolutionary History, Transcriptome Expression Profiles, and Abiotic Stress Responses of the *SBP* Family Genes in the Three Endangered Medicinal *Notopterygium* Species

**DOI:** 10.3390/ijms27020979

**Published:** 2026-01-19

**Authors:** Dan-Ting Zhang, Yan-Jun Cheng, Rui Yang, Hui-Ling Wang, Xiao-Jing He, Cai-Yun Luo, Zhong-Hu Li, Mi-Li Liu

**Affiliations:** 1 Key Laboratory of Resource Biology and Biotechnology in Western China, Ministry of Education, College of Life Sciences, Northwest University, Xi’an 710069, China; 2 College of Advanced Agricultural Sciences, Yulin University, Yulin 719000, China

**Keywords:** *SBP* gene family, *Notopterygium*, gene structure, phylogenetic analysis, population evolution, abiotic stresses

## Abstract

Squamosa promoter binding protein (SBP) plays a vital role in plant growth, development, and responses to abiotic stresses. The genus *Notopterygium* is an endangered perennial herbaceous plant mainly distributed in the high-altitude Qinghai–Tibet Plateau and adjacent areas, which possibly occurred the adaptive evolution to the extreme environmental conditions. In this study, we firstly determined the genome-wide structural characteristics, evolutionary history, and expression profiles of the *SBP* family genes in *Notopterygium* species by using genome, transcriptome, and DNA resequencing data. We have also investigated the response patterns of *SBPs* of *N. franchetii* to the drought and high-temperature stresses. The 21, 18, and 18 *SBP* family genes of three *Notopterygium* species, *N. incisum*, *N. franchetii*, and *N. forrestii*, were, respectively, identified and classified into eight subfamilies, with four subfamily members regulated by miR156. The structure analysis showed that the members of the same *SBP* subfamily had similar structures and conserved motif composition. *Cis*-element analysis suggested that those *SBP* genes may have been essential to the growth and environmental adaptation of *Notopterygium*. The expansion of the *SBP* gene family was mainly caused by the whole genome duplication/segmental duplication and transposable element duplication. Evolutionary analysis showed the *SBP* gene family experienced severe contraction events and most of the gene copies underwent purification selection. Population genetics analysis based on *SBPs* variations suggested that the genus *Notopterygium* species have obvious genetic structure and interspecific differentiation. RNA-seq and qRT-PCR experiments demonstrated that the expressions of *SBPs* genes in *Notopterygium* were not species-specific, but tissue-specific. *NinSBP08* and *NinSBP10/12* may have played the key roles in heat tolerance and drought resistance, respectively. These results provided novel insights into the evolutionary history of the *SBP* gene family in the endangered herb *Notopterygium* species in the high-altitude Qinghai–Tibet Plateau and adjacent areas.

## 1. Introduction

Squamosa promoter binding protein (SBP/SPL) is a unique transcription factor in plants. The *SBP* gene family has a conserved domain composed of 74–79 highly conserved amino acid residues, including two zinc finger structures and a nuclear localization signal. The first zinc finger has the following two types: Cys-Cys-Cys-His (C3H) and Cys-Cys-Cys-Cys (C4). The second zinc finger is usually Cys-Cys-His-Cys (C2HC). There is a partial overlap between the C-terminal nuclear localization signal and the second zinc finger structure, which guides SBP transcription factors into the nucleus and then regulates the expression of downstream genes [1]. The *SBP* gene was first isolated from *Antirrhinum majus* L. in 1996 [2]. Due to its ability to recognize the binding site of the flower development gene SQUAMOSA promoter and regulate gene expression, it was named the SBP transcription factor [2]. Subsequently, the *SBP* gene was identified in many angiosperm plants, such as *Chlamydomonas reinhardtii* Karl Friedrich Reinhardt (7), *Physcomitrella patens* (Hedw.) Bruch & Schimp. (13), *Populus trichocarpa* Torr. & Gray (28), *Arabidopsis thaliana* (L.) Heynh. (16), *Mangifera indica* L. (26), *Vitis vinifera* L. (18), and *Dactylis glomerata* L. (17) [3,4,5,6,7,8,9]. As the number of *SBP* genes isolated from different green plants increased, researchers conducted the evolutionary analysis of this gene family. It has been shown that the *SBP* gene family originated from the differentiation of green algae and terrestrial plant ancestors, followed by further duplication and differentiation in each lineage, including the process of exon–intron loss [4]. Some studies showed that the *SBP* gene had undergone both ancient and recent duplication events, leading to the formation of *SBP* homologous genes in most of plants [10,11,12,13]. The *SPL* gene family of *Arabidopsis thaliana* (*AtSPLs*) were classified into eight subgroups based on the phylogenetic analysis, where each group shared similar motifs structures and conserved motif composition [14]. Some studies found that the replicated *SBP* gene was maintained in the genome by positive selection after subfunctionality and new functionality [15].

Most *SBP* genes play important roles in plant growth and development through targeted regulation of miR156, such as growth plasticity [15], root development and nodulation [16], leaf formation [17], flowering regulation [18,19], fruit development, and seed growth [20,21]. Furthermore, more and more *SBP* genes have been reported in responses to various biotic and abiotic stresses. SPL transcription factors were potential targets for epigenetic regulation by chickpea (*Cicer arietinum* L.) under drought stress [22]. *OsSPL10* (*Oryza sativa SPL10*) knockout mutants enhanced drought tolerance by inducing rapid stomatal closure and preventing water loss [23]. The *OfSPL11* (*Osmanthus fragrans SPL11*) transgenic line had a faster germination rate, longer roots, and less leaf wilt than the wild type under salt stress [24]. *SPLs* could regulate cold tolerance in plants by mediating the *CBF*-mediated cold signaling pathway [25,26]. In alfalfa, the resistance to heat stress (40 °C) was increased in *SPL13* knockout plants [27]. In addition, SBP transcription factors may be involved in glutathione-mediated pesticide degradation in tomato (*Solanum lycopersicum* L.) [28]. *SPL10* could enhance both the activity of salicylic acid pathway by directly activating *PAD4* and the age-related resistance of *Arabidopsis* [29].

The genus *Notopterygium* H. Boissieu species are endangered perennial herbaceous plants endemic to China, belonging to the Apiaceae family. *Notopterygium* plants are mainly distributed in the Qinghai–Tibet Plateau (QTP) and adjacent areas [30,31]. Influenced by the recent uplift on the eastern margin of the QTP, *Notopterygium* species diverged approximately 1.74–7.82 million years ago [32]. Some studies suggested that the genus *Notopterygium* should have been clustered into four species clades, *N. incisum* C. C. Ting ex H. T. Chang, *N. franchetii* H. de Boissieu, *N. forrestii* H. Wolff, and *N. oviforme* R. H. Shan [33]. Among them, *N. incisum* and *N. forrestii* formed separate branches, and *N. franchetii* and *N. oviforme* clustered into sister branches [34,35]. Moreover, the genetic differentiation coefficient between *N. incisum* and the other three species was the largest, followed by *N. forrestii* and *N. franchetii*; *N. franchetii* and *N. oviforme* were the smallest [32]. Furthermore, both *N. forrestii* and *N. oviforme* were formed by species hybridization. The hybridization of *N. incisum* and *N. franchetii* formed *N. forrestii*, and *N. forrestii* backcrossed with *N. franchetii* produced *N. oviforme* [30]. *N. incisum* and *N. franchetii* are the source plants of *Notopterygii* Rhizoma et Radix (NRR) included in the Chinese Pharmacopeia, which are used as medicine with their dry roots and rhizomes [36].

In this study, we identified *SBP* family genes from the whole genomes of *N. incisum*, *N. franchetii*, and *N. forrestii*. The gene characteristics, structural composition, subcellular localization, and promoter *cis*-regulatory element were systematically analyzed. Meanwhile, the SBP transcription factor families of *Daucus carota* L., *Coriandrum sativum* L., *Apium graveolens* L., *Angelica sinensis* (Oliv.) Diels, and other representative species in Apiaceae were analyzed jointly, and their phylogenetic relationships and evolution were calculated. Then, the genetic variation and population structure of *SBP* gene in *Notopterygium* species were detected based on the population genome resequencing data. Finally, the expression patterns of *SBP* gene in *Notopterygium* species under different tissue stages and environmental stresses were investigated by using RNA-seq data and real-time quantitative PCR. This study will provide a theoretical basis for the development of specific gene functions of *Notopterygium* and the breeding of new resistant varieties.

## 2. Results

### 2.1. Identification and Characterization of SBP Family Genes

A total of 21, 18, and 18 *SBP* family genes were identified from the whole genomes of *N. incisum*, *N. franchetii*, and *N. forrestii*, respectively. They were renamed *NinSBP01-NinSBP21*, *NfrSBP01-NfrSBP18*, and *NfoSBP01-NfoSBP18* according to their position on the chromosome, respectively. The *SBP* genes were unevenly distributed on the chromosomes, with none of the genes localized on chr3 and chr7 in any of the three *Notopterygium* species. In addition, *NfrSBP16*, *NfrSBP17*, and *NfrSBP18* did not map to any of the chromosomes (Figure S3). Meanwhile, 19, 23, 15, 20, 20, and 18 members of the *SBP* gene family were identified from the genomes of carrot, coriander, celery, *A. sinensis*, *A. elata*, and grape (Figure S4). They were renamed *DcSBP01-DcSBP19*, *CsSBP01-CsSBP23*, *AgSBP01-AgSBP15*, *AsSBP01-AsSBP20*, *AeSBP01-AeSBP20*, and *VvSBP01-VvSBP18* in the same method (Figure S5).

The sequence length of SBP protein of the three *Notopterygium* species was from 136 to 1095 amino acids, with corresponding molecular weights of 15.87–120.37 kDa, isoelectric points of 5.74–9.7, and basic proteins accounting for 84%. The results of subcellular localization showed that *NinSBP04*, *NfrSBP06*, *NfoSBP05*, and *NfoSBP11* were localized to plasma membrane and nucleus, and the rest were localized to nucleus (Table 1). In order to verify the accuracy of subcellular localization, the DeepTMHMM tool was used for transmembrane domain detection. *NinSBP04*, *NfrSBP06*, *NfoSBP05*, and *NfoSBP11* proteins all had transmembrane domains, consistent with the results of subcellular localization (Figure S6).

### 2.2. Phylogenetic Analysis

**Figure 1:**
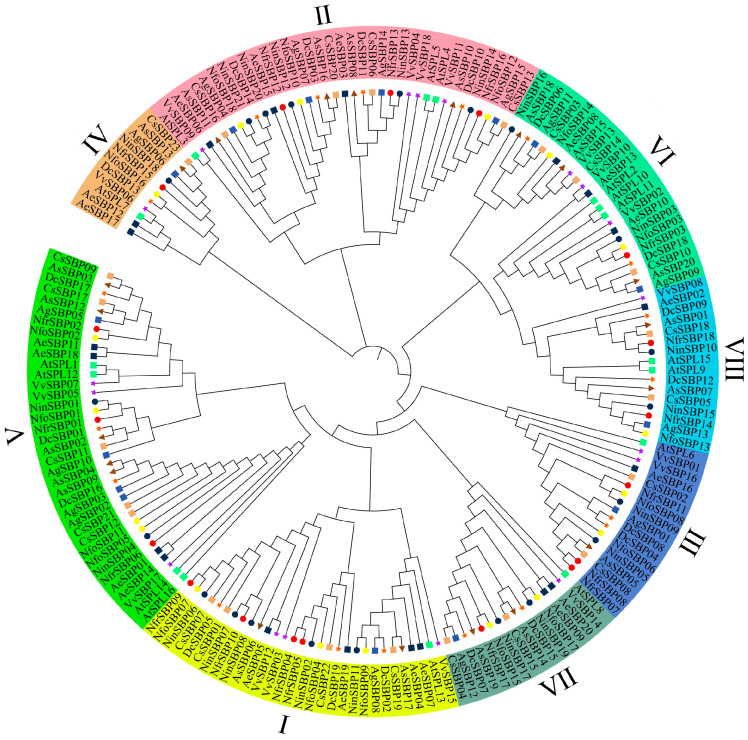
The ML phylogenetic tree constructed from conserved domain sequences of *SBP* family genes in the three *Notopterygium* species (*N. incisum*, *N. franchetii*, and *N. forrestii*), and carrot, coriander, celery, grape, *A. sinensis*, *A. elata*, and *A. thaliana*, and all members of the *SBP* family genes were classified into 8 subfamilies. The differently shaped symbols preceding the gene IDs represent different species, with circles denoting the three species of *Notopterygium*. Orange pentagrams represent carrots, and purple pentagrams represent grapes. Dark blue, green, light brown, and black squares represent *Apium graveolens*, *Arabidopsis thaliana*, *Coriandrum sativum*, and *Aralia elata*, respectively; brown triangles represent *Angelica. sinensis*.

To explore the phylogenetic relationships of *SBP* family genes in *Notopterygium* species, we constructed ML phylogenetic trees with 188 SBP proteins from ten species (Figure 1). According to the classification of *AtSPL* genes, the *SBP* family genes of all species were classified into eight subfamilies, which indicated that the evolution of *SBP* genes was relatively conservative. Multiple sequence alignment results showed that the first zinc finger structure of all members in subfamily IV was C4 type, while the other subfamilies were C3H type (Figures S2 and S4). For *Notopterygium* species, subfamilyIhad the most members, with a total of twelve genes. Meanwhile, the subfamily IV had the fewest, with only three members (Figure 1).

### 2.3. SBP Protein Tertiary Structure

**Figure 2:**
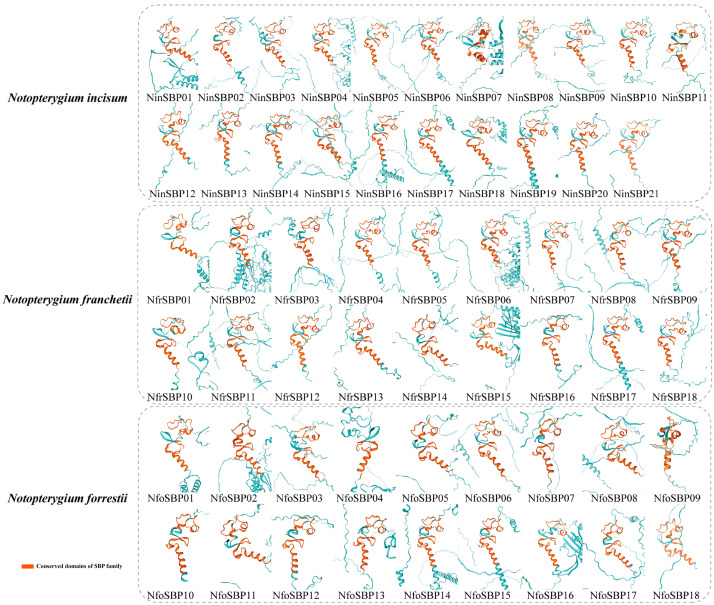
Three-dimensional structures of SBP protein in the three *Notopterygium* species.

Protein structure often correlates its function. The results of homology modeling using the SWISS-MODEL are shown in Figure 2. SBP proteins of the same subfamily had similar three-dimensional structures. For example, all members of subfamily V had more complex tertiary structures, while all members of subfamily IV had a similar structure that differ from members of other subfamilies. Therefore, it was inferred that members of the same subfamily have similar functions.

### 2.4. Characterization of Gene Structure and Conserved Motifs

**Figure 3:**
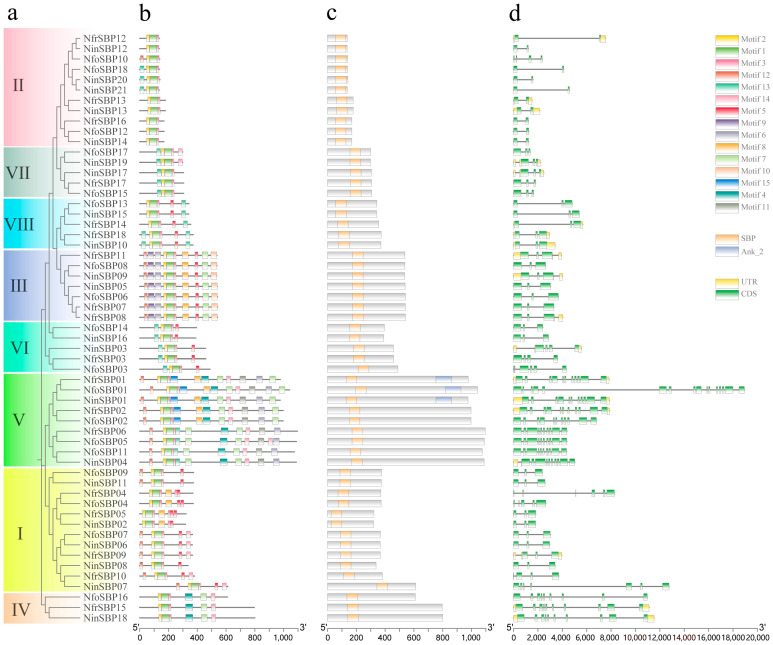
The phylogenetic tree, gene structure, conserved domains, and conserved motifs of *SBP* family genes in the three *Notopterygium* species. (**a**) The ML tree was constructed based on the sequences of conserved domains of SBP proteins using MEGA software (version 11.0.13); (**b**) the conserved motifs of *SBP* family genes; (**c**) the conserved domains of SBP proteins; (**d**) and the structure of *SBP* family genes.

The gene structures, conserved domains, and conserved motif composition of *NinSBPs*, *NfrSBPs*, and *NfoSBPs* are shown in Figure 3. All identified protein sequences contained intact SBP-conserved domains, namely two zinc fingers and one nuclear localization signal. Phylogenetic analysis of *NinSBPs-*, *NfrSBPs-*, and *NfoSBPs-*conserved domain sequences was performed (Figure 3a); its topology was consistent with the phylogenetic tree constructed in ten species (Figure 1). As shown in Figure 3c, in addition to the SBP-conserved structural domain, *NinSBP01*, *NfrSBP01*, and *NfoSBP01* also contained the Ank_2 conserved domain at the C-terminus.

The MEME results showed that, corresponding to the conserved domain, all members contain motifs 1, 2, and 3 (Figure 3b and Figure S7). In terms of the number of motifs, subfamily V contained the highest number of motifs (11–12), followed by subfamily III, with all members comprising 10 motifs. The subfamily II included the lowest number of motifs (3–4), while other subfamilies had 4–6 motifs. From the analysis of motif composition, some motifs were unique to a certain family, such as motif 9/10 which were only distributed in subfamily III, and motif 11/15 were only distributed in subfamily V. Some motifs were shared by different subfamilies, such as motif 5 in subfamilies I, III, V, VI, and VIII, and motif 7 only in subfamilies III, IV, and V. Therefore, different subfamilies may be functionally similar, but not identical.

The exon/intron distribution of *NinSBPs*, *NfrSBPs*, and *NfoSBPs* was analyzed by the GSDS2.0 program (Figure 3d). The distribution patterns of the same subfamily in terms of exon length and intron number were roughly similar. Corresponding to the conserved motifs, subfamily V had the highest number of exons/introns with 10–16/9–15 (Table 1, Figure 3d), followed by subfamily IV with 9–10/8–9, whereas subfamily II had the lowest exon/intron members (2/1). This was probably due to the deletion of introns at the late stage of differentiation. The difference in exon/intron numbers in different subfamilies indicated that different selection pressures may have been experienced during evolution, leading to functional differentiation.

### 2.5. Cis-Element Analysis

**Figure 4:**
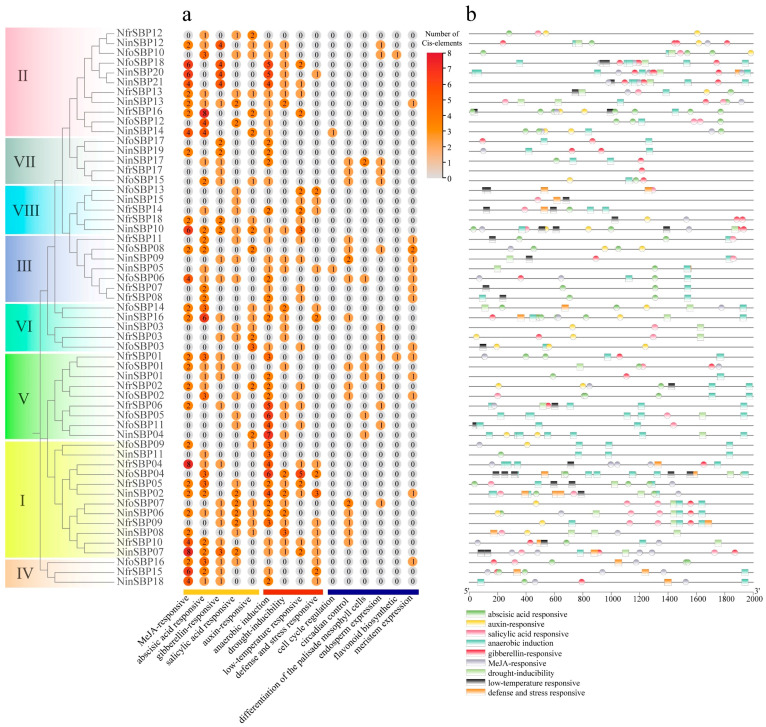
*Cis*-elements of *SBP* genes promoter in the three *Notopterygium* species. (**a**) The number of *cis*-acting elements in each *SBP* gene promoter region; (**b**) the distribution of *cis*-acting elements related to hormone and environmental response.

A total of twenty-three regulatory elements related to environmental stress, hormone response, and growth and development were detected in *SBP* gene promoters of the three *Notopterygium* species, corresponding to fifteen functions (Figure 4a). Among them, all genes contained the most hormone-responsive elements (288), followed by environmental stress-responsive elements (200), and the least growth-related elements (60). To further analyze the response of *SBP* family genes to environment and hormones, we constructed an environmental- and hormone-response element map (Figure 4b). The number and distribution of the same stress-response elements varied greatly among subfamilies. For example, most of the members contained anaerobic induction elements, but only nineteen members included stress and defense response elements. The differences in the number and function of these elements suggest that *SBP* genes in *Notopterygium* plants may be involved in various biotic/abiotic stresses and the regulation of environmental adaptation.

### 2.6. miRNA156-Targeting SBPs and Protein Interaction Network

In order to further explore the regulatory mode of *SBP* gene in *Notopterygium* plants, miR156 regulatory site detection was performed on *SBP* family genes of three *Notopterygium* species, and the results are shown in Table 2. miR156 regulatory sites were detected in 29 out of 57 *SBP* gene family members from the three *Notopterygium* species. Among them, miR156 regulatory sites were detected in 11 genes from *N. incisum*, 10 and 8 in *N. franchetii* and *N. forrestii*, respectively. These *SBP* genes belong to subfamily I, III, VI, and VIII, respectively (Table 2).

Except for *NinSBP03/07*, most SBP proteins were involved in different regulatory networks (Figure S8). It can be observed that SBP proteins interact with a large number of flowering-related proteins, e.g., AP2, TOE, AGL, LFY, SOC1, SMZ, and RGA. In addition, part of *NinSBPs* were associated with abiotic stress-related proteins such as MYB, TCP, NBR1, and RAP2-7. These results suggest that SBP proteins in *Notopterygium* species interact with flowering and abiotic stress-related proteins and thus participate in plant growth and development.

### 2.7. Collinearity and Gene Replication Types

**Figure 5:**
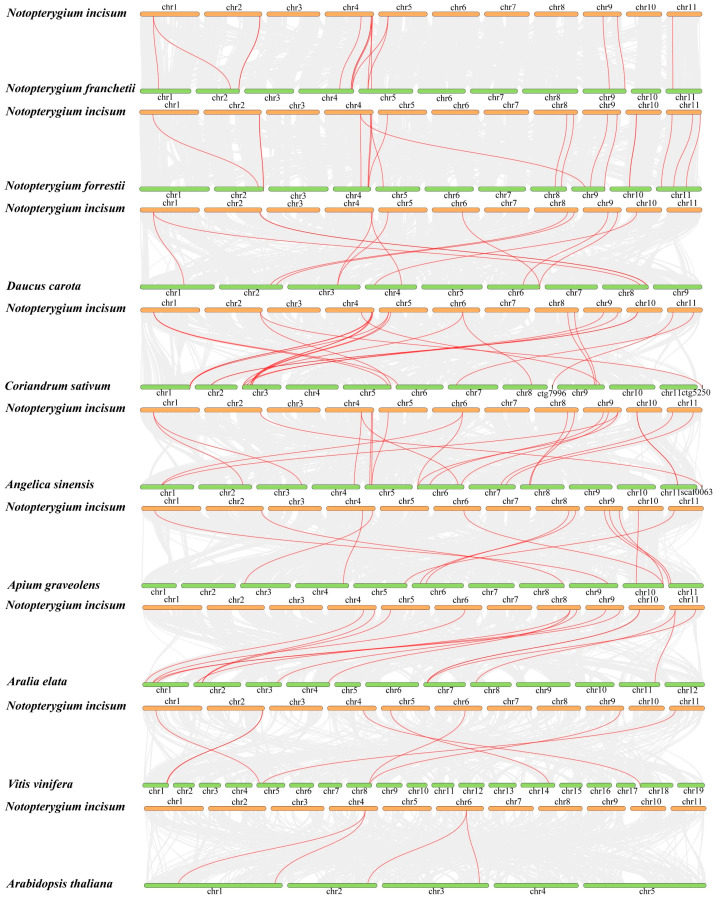
The synteny analysis of *NinSBPs* with *NfrSBPs*, *NfoSBPs*, *DcSBPs*, *CsSBPs*, *AsSBPs*, *AgSBPs*, *VvSBPs*, and *AtSBPs*, respectively. The gray lines represent collinearity blocks between genomes, while the red lines represent *SBP* collinearity genes.

There were collinear gene pairs between *NinSBPs* and *SBP* genes of other species, 18 (*NfoSBPs*), 15 (*NfrSBPs*), 13 (*DcSBPs*), 22 (*CsSBPs*), 20 (*AsSBPs*), 12 (*AgSBPs*), 14 (*AeSBPs*), 8 (*VvSBPs*), and 4 (*AtSBPs*) collinear gene pairs, respectively (Figure 5). We found that, except for *NinSBP20*, other *NinSBPs* genes covary with at least one other species, such as *NinSBP04* which covaries with *AtSPL14*, *VvSBP17*, *AeSBP01*, *AsSBP04*, etc. This indicated that these *NinSBPs* genes were relatively conserved before differentiation. In addition, there was a collinear relationship between *NinSBPs* and 2–3 *SBP* genes from another species, such as *NinSBP05* and *NfoSBP06/08*, *NinSBP01* and *DcSBP01/17*, suggesting that these genes play important roles in species evolution and the execution of biological functions.

Gene replication-type analysis was performed using the duplicate_gene_classifier program of MCScanX software (version 11.0.13) (Figure S9a). Except for three *NinSBPs* genes that belong to proximal duplication, all other genes were WGD/segmental duplication and transposable element duplication types, without tandem duplication. Two, four, and four paralogous gene pairs of *N. incisum*, *N. franchetii*, and *N. forrestii* were all the results of segmental duplication. Among them, eight paralogs of the three species belong to subfamilies I, III and V, respectively (Figure S9b–d).

### 2.8. Ka/Ks and Gene Duplication/Losses

**Figure 6:**
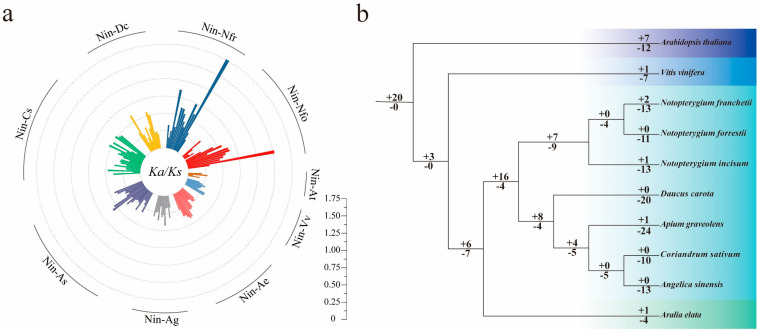
*Ka/Ks* value and the duplication and loss analyses. (**a**) The *Ka/Ks* calculations of the orthologous *SBP* gene pairs between *N. incisum* and the other 9 species; (**b**) the duplication and loss analyses of the *SBP* family genes in the 10 species, where “+” and “–” indicate duplication and loss, respectively, and the number after “+” and “–” represents the gene number.

To determine the nature and extent of selection pressure on repetitive gene pairs, *Ka*, *Ks*, and *Ka/Ks* values were calculated for 125 homologous gene pairs in ten species. The results showed (Figure 6a) that the *Ka/Ks* values of *NinSBP02/NfrSBP04* and *NinSBP02/NfoSBP04* were 1.51 and 1.27, respectively. It suggested that non-synonymous substitutions of some genes during the differentiation process were beneficial to the species and were preserved by positive environmental selection. However, the *Ka/Ks* values of the other homologous gene pairs were all less than 1, and 105 of them were less than 0.50. Therefore, they all underwent purification selection during evolution. In addition, the *Ka/Ks* values of the paralogue gene pairs in the three *Notopterygium* species were also less than one (Table S1).

The *SBP* genes of ten species had a total of 77 gene duplicates and 165 gene losses during evolution, with the number of gene losses far greater than duplicates (Figure 6b). Moreover, the common ancestor of all species had twenty gene duplicates without loss, and the number of gene duplications in the common ancestor lineage of six Apiaceae species was four times the number of gene losses. It indicated that *SBP* gene family experienced large-scale expansion in the early stage of species differentiation. Compared with other genera in Apiaceae family, the number of *SBP* gene losses in the common ancestor lineage of *Notopterygium* plants was greater than the number of duplicates, and the overall number of *SBP* genes was significantly reduced. Furthermore, all species had a large number of gene loss; presumably, *SBP* gene family has experienced a serious contraction in recent evolutionary processes.

### 2.9. Genetic Diversity and Population Genetic Structure

A total of 20,727 SNPs were detected, and 525 high-quality SNPs were obtained after filtering. Annotation results for SNPs showed that the majority of SNPs were located in exonic regions (40.32%), followed by gene downstream regions (28.09%) and intronic regions (26.21%). The splice site region and 5′ untranslated region were the fewest (less than 1%) (Table 3). Then, 156 SNPs were annotated as synonymous mutations, 145 as missense mutations and one stop codon mutation. In addition, SNPs were unevenly distributed on the chromosome, with 54.86% of SNPs positioned on chr4 (Table S2).

**Figure 7:**
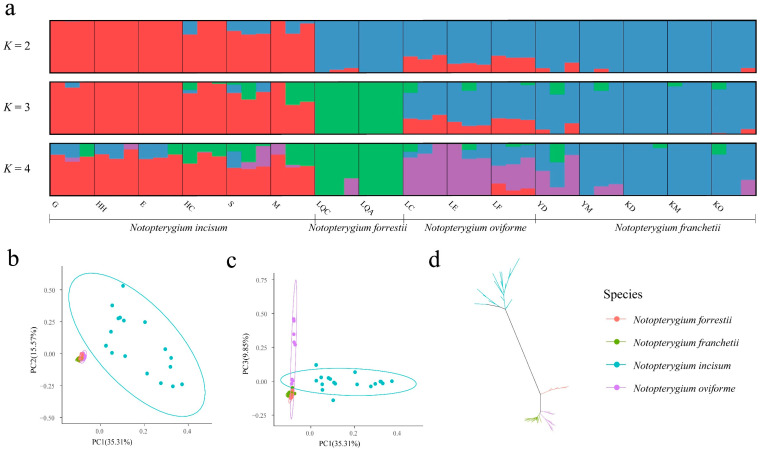
The population structure of *SBP* gene family in *Notopterygium* species. (**a**) Genetic structure analysis; (**b**,**c**) principal component analysis, and the confidence interval is 95%; and (**d**) ML tree.

The population genetic structure calculated based on ADMIXTURE software (version 1.3.0) showed that the optimal cluster of *SBP* gene family in *Notopterygium* species was three. When *K* = 2, the genetic component of *SBP* genes in *N. incisum* was the first to be isolated from the *Notopterygium* population; when *K* = 3, the genetic component of *SBP* genes in *N. forrestii* was subsequently isolated; and when *K* = 4, there was a clear distinction between the *N. franchetii* and *N. oviforme* populations, but some populations had more gene introgression (Figure 7a). Both PC1-PC2 and PC1-PC3 showed significant genetic differentiation between the *SBP* gene family of *N. incisum* and the other three species, consistent with the results of ADMIXTURE (Figure 7b,c). An ML tree was constructed using SNPs (Figure 7d). The *SBP* genes of *N. incisum* and *N. forrestii* were clustered into a separate genetic branch, respectively. The *SBP* genes of 9 individuals in *N. oviforme* were classified into two genetic branches, and 6 of them and 15 individuals of *N. franchetii* were grouped into a large branch. This indicated that the genetic differentiation of the *SBP* genes in *N. incisum* and *N. forrestii* was relatively large, while that in *N. franchetii* and *N. oviforme* was relatively small.

The genetic differentiation index (*F*st) of *SBP* gene family between *Notopterygium* species is shown in Figure S10. The *F*st was the largest between the *SBP* genes of *N. incisum* and *N. franchetii*, followed by those between *N. incisum* and *N. oviforme*, *N. incisum* and *N. forrestii*, *N. forrestii* and *N. franchetii*, *N. forrestii* and *N. oviforme*, and the smallest between *N. franchetii* and *N. oviforme*, with values of 0.30, 0.22, 0.20, 0.10, 0.08, and 0.07, respectively. Within-species nucleotide diversity indicated that the *SBP* family genes of *N. oviforme* had the highest nucleotide diversity level (*θ*π = 7.84 × 10^−4^), followed by *N. forrestii* (*θ*π = 7.56 × 10^−4^) and *N. franchetii* (*θ*π = 7.05 × 10^−4^), and the *N. incisum* had the lowest (*θ*π = 6.37 × 10^−4^).

### 2.10. Expression Profiles of SBP Gene Family

**Figure 8:**
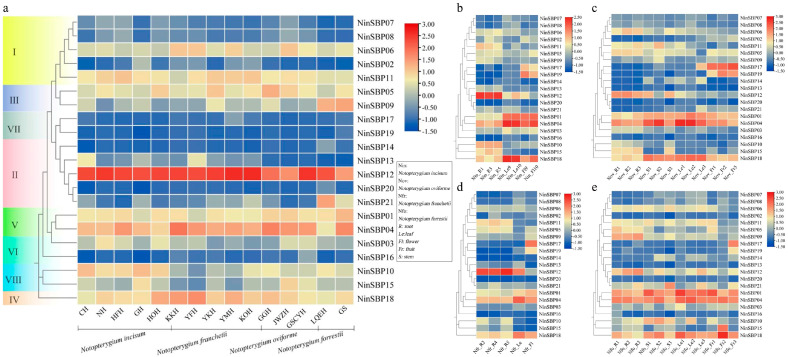
Expression patterns of the *NinSBPs* gene. (**a**) Expression patterns of the *NinSBPs* gene in the root tissues of the four *Notopterygium* species; the abbreviations on the horizontal axis of the heatmap correspond to 15 populations; (**b**–**e**) expression patterns of the *NinSBPs* gene in different tissues of *N. incisum* (**b**), *N. oviforme* (**c**), *N. franchetii* (**d**), and *N. forrestii* (**e**).

In order to investigate the expression patterns of *SBP* genes in *Notopterygium* species, we constructed expression heatmaps of different tissues (Figure 8). Most genes had similar expression levels in roots of different species, for example, *NinSBP12* was most highly expressed in all populations of the four species, while *NinSBP17* and *NinSBP19* were not expressed in all populations. Only a few genes were expressed differently among species, such as *NinSBP09*, which was more expressed in *N. incisum* than in the other three species (Figure 8a). These results indicated that the expression level of *NinSBPs* varied among species depending on the gene members. In addition, the expression levels of genes in the same subfamily were not completely similar, such as the significant difference in *NinSBP12* and *NinSBP20*, which were all located in the second subfamily. Therefore, there was no correlation between gene expression levels and subfamily.

### 2.11. qRT-PCR

**Figure 9:**
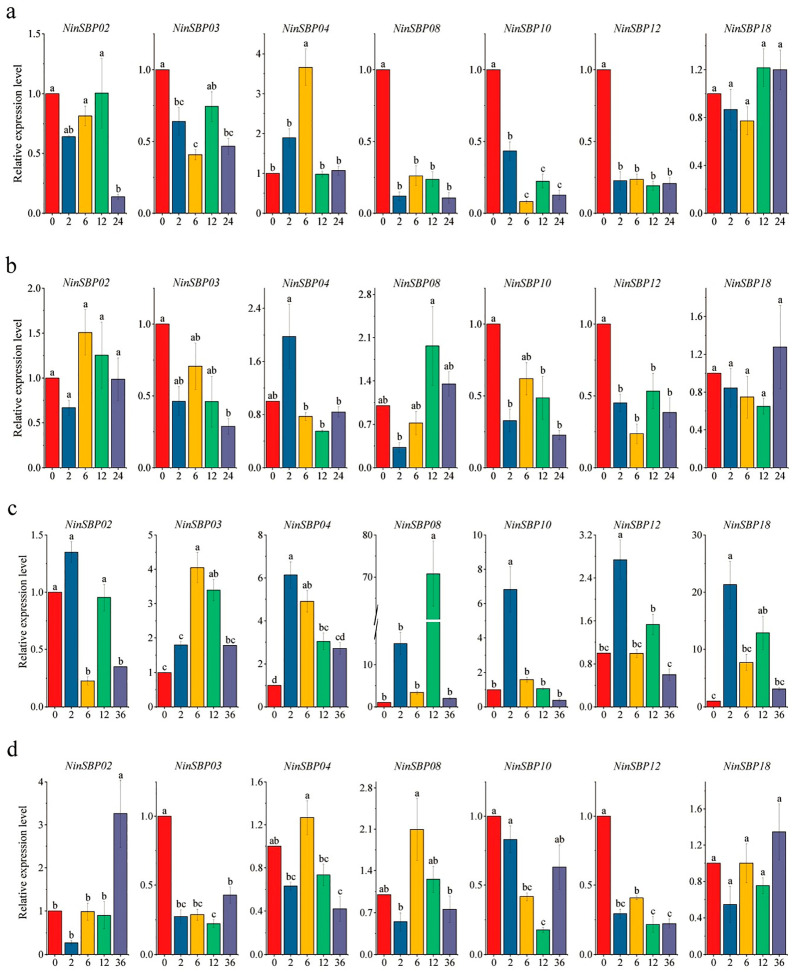
Relative expression levels of *NinSBPs* in leaves and roots of *N. franchetii* under drought and high temperature stresses. (**a**) Leaves under drought stress; (**b**) roots under drought stress; (**c**) leaves under high temperature stress; and (**d**) roots under high temperature stress. Note: Bars sharing the same letter are not significantly different; different letters denote significant differences (*p* < 0.05).

To investigate the response of *SBP* genes in *Notopterygium* plants to drought and high temperature stresses, qRT-PCR experiment was carried out. Different *NinSBPs* genes exhibited different response patterns under the same abiotic stress (Figure 9).

Under drought stress, expression of several *NinSBP* genes in leaves showed dynamic changes. *NinSBP02*/*18* exhibited a transient induction, peaking at 12 h. In contrast, *NinSBP03*/*08*/*12* showed highest expression at 0 h. *NinSBP04* displayed a distinctive bimodal response (increase–decrease–increase), peaking at 6 h, while *NinSBP12* expression decreased initially and then stabilized at 2 h (Figure 9a).

In roots, the expression trends of *NinSBP02*/*03*/*04*/*08* were consistent with those in leaves, albeit with shifted peak times (6, 0, 2, and 24 h, respectively). *NinSBP10/12* in roots showed a similar response pattern to that in leaves, peaking at 0 h. Conversely, *NinSBP18* in roots displayed a relatively simple increase in stress responsiveness, reaching its maximum at 24 h (Figure 9b).

Under high temperature stress, there were two trends of *NinSBPs* expression in leaves of *N. franchetii* (Figure 9c,d). One group (*NinSBP02*/*08*/*12*/*18*) showed complex fluctuations, with most (*NinSBP02*/*12*/*18*) peaking early at 2 h, while *NinSBP08* peaked later at 12 h. The other group (*NinSBP03*/*04*/*10*) consistently showed a rapid induction followed by a gradual decline, peaking at 6 h *(NinSBP03*) and 2 h (*NinSBP04/10*) (Figure 9c). Root responses were more varied: *NinSBP02*/*03*/*12*/*18* fluctuated with peaks at 36 h *(NinSBP02*/*18*) and 0 h (*NinSBP03*/*12*). *NinSBP04*/*08* exhibited a decrease–increase–decrease pattern, peaking at 6 h. Only *NinSBP10* showed a simple decrease–then–increase trend, with a maximum at 0 h (Figure 9d). Furthermore, Figure S11 illustrated the antioxidant enzyme activities in the roots and leaves of *N. franchetii* at various time points under the aforementioned different stress treatments.

## 3. Discussion

### 3.1. Identification and Structure Analysis of SBP Gene Family

*Notopterygium* is an endangered and medicinal herb genus endemic to China, and the *N. incisum* and *N. franchetii* of this genus have high medicinal and economic values. The whole genome sequencing of *Notopterygium* species provided an opportunity for the evolutionary study of *SBP* gene family. Then, 21, 18 and 18 members of *SBP* gene families were identified from *N. incisum*, *N. franchetii*, and *N. forrestii*, respectively. Furthermore, we identified 19, 23, 15, 18, 20, and 20 *SBP* genes in the genomes of carrot, coriander, celery, grape, *A. sinensis*, and *A. elata*, respectively, for a comparative analysis with *Notopterygium* species. The number of *SBP* genes in different species may be related to the amount of loss and retention after multiple whole genome duplication events [37]. Consistent with findings from other species, the distribution of *SBP* genes in *Notopterygium* species was uneven on chromosomes [38,39]. *NfrSBP16*/*17*/*18* was not localized to any chromosome, but *NfrSBP16*/*17*/*18* shares similar gene structures and conserved motif compositions with other members of its subfamily, and these genes all have orthologous genes in the *SBP* gene of *N. incisum.* It is therefore likely that *NfrSBP16*/*17*/*18* was indeed part of the genome, although the exact chromosomal location in the current assembly has not yet been determined. In addition, subcellular localization results revealed that *NinSBP04*, *NfrSBP06*, *NfoSBP05*, and *NfoSBP11* were localized to the plasma membrane and possessed transmembrane domains, in contrast to the predicted nuclear localization of most SBP proteins. This finding was consistent with observations in *Zanthoxylum bungeanumn* [40]. Such atypical characteristics suggest functional diversity within the *SBP* gene family. For instance, the *Arabidopsis* SPL7 protein contains a functional transmembrane domain and exhibits dual localization in the nucleus and the membrane system, which is associated with its role in responding to copper deficiency [41]. We speculate that these membrane-associated SBP proteins in *Notopterygium* species may act as sensors or regulatory nodes for environmental signals, participating in membrane-related stress-response pathways.

The results of gene structure prediction of *SBP* family genes in the three *Notopterygium* plants showed that members of the same subfamily have similar tertiary protein structures, conserved motifs, and exon/intron composition patterns. It is therefore presumed that they share a common evolutionary origin and similar molecular functions. The tertiary structure of the fifth subfamily was more complex and diverse, with the highest number of introns (9–15). Introns increase the length and frequency of gene recombination and alter their regulatory role [42]. Therefore, we speculate that this subfamily has significant functional differentiation and may perform particular biological functions under certain specific conditions. It is generally accepted that a large number of introns were present in ancient biological ancestors but were lost as organisms evolved [42,43]. It is inferred that the *SBP* gene of subfamily V was relatively old, while the subfamily II was relatively late. In addition, the protein tertiary structure of subfamily IV differs greatly from that of other subfamily members, possibly because the first zinc finger structure was type C4. According to the evolutionary model of the *SBP* gene family proposed by Guo et al. (2008), this subfamily originated from group I [4].

Promoter *cis*-acting elements can provide insights into the function of genes [44]. This study suggests that the *SBP* genes promoter of *Notopterygium* species contained several *cis*-acting regulatory elements, which may regulate a variety of developmental and stress-related processes. However, different *SBP* genes included different regulatory elements, which may be closely related to the functional diversity of *SBP* genes. In addition, a large number of anaerobic response elements suggest that *SBP* gene may play an important role in plant adaptation to high-altitude oxygen-poor environment.

### 3.2. Evolutionary History of SBP Gene Family in Notopterygium

The *SBP* gene originated from the differentiation between green algae and terrestrial plants and subsequently underwent further duplication and differentiation in each lineage [4]. In this study, ML phylogenetic tree was constructed using conserved domain sequences of SBP proteins from ten species. *SBP* genes of all species were classified into eight subfamilies. The gene structure and conserved motifs of *NinSBPs*, *NfoSBPs*, and *NfrSBPs* also supported this taxonomic pattern, suggesting that the *SBP* gene family was highly conserved during evolution and had similar biological functions in different species. The results of collinearity analysis showed that the *SBP* gene of *N. incisum* had more collinearity gene pairs with the species of Apiaceae and Araliaceae, but less with Vitaceae and Brassicaceae, which also confirmed the relationship between the species. *NinSBP20* had no collinearity with any *SBP* gene. *NinSBP21* only had collinearity with *NfoSBP18*, while its *Ks* value was zero. The phylogenetic tree showed that these three genes were located in the same branch of subfamilies II; it is inferred that these three genes are highly homologous and have been differentiated recently. Furthermore, *NinSBP10* has a collinearity with *SBP* genes of all species except *Notopterygium* plants; it is hypothesized that *NinSBP10* was relatively old and underwent great variation after *Notopterygium* differentiation.

Repeated genes are generally considered to be an essential material source for the origin of new species [45]. In this study, only WGD/segmental duplication, dispersed duplication, and proximal duplication were detected in *NinSBPs*, *NfrSBPs*, and *NfoSBPs*, and no tandem duplication events were present. This phenomenon has also been found in beet (*Beta vulgaris* L.) [46]. These results suggest that some *SBP* genes may be generated during WGD/segmental duplication, especially members of subfamilies III and V, leading to genomic complexity [47]. Additionally, these replication events may be driving factors for the new functions of *SBP* genes, helping plants adapt to harsh environments [11,45]. In addition, 50% of the *SBP* paralogous genes within *Notopterygium* species were located on chr4 and chr5, respectively, and 54.86% of SNPs were distributed on chr4 (Table 4). It is inferred that there may be a chromosomal rearrangement between chr4 and chr5 and that this region was selected conservatively during evolution.

Genome-wide duplication contributes greatly to the evolution of eukaryotic genomes; meanwhile, WGD events accelerate gene loss [48,49]. Similarly to other species in the Apiaceae family, the *SBP* gene in *Notopterygium* plants has undergone a large amount of gene loss, presumably caused by two consecutive WGD events unique to Apiaceae [50,51,52]. *Ka/Ks* calculations showed that the vast majority of *SBP* genes in *Notopterygium* plants have undergone purification selection. Meanwhile, only two (1.6%) duplicate gene pairs were detected by positive selection, indicating that they may have undergone relatively rapid evolution. Therefore, the *SBP* gene in *Notopterygium* may have limited functional differentiation after WGD [53].

### 3.3. Genetic Diversity and Population Structure of SBPs

Based on comparative reference genomes and whole-genome resequencing data, a large number of genetic features such as single nucleotide polymorphism variations, insertion and deletion variations, structural variations, and copy number variations can be obtained to explain genetic variation patterns at the genome level [54]. In this study, SNPs data of forty-eight individuals from fifteen populations of four species in *Notopterygium* were used to analyze the genetic diversity and genetic structure of *SBP* genes. The annotation results of the SNPs revealed that 40.32% of the variant sites were located in exonic regions. Those SNPs lead to the changes in the codon and amino acid sequence, resulting in changes in the structure and function of the proteins, ultimately bringing about functional diversity of the *SBP* gene in *Notopterygium* species.

The findings of population genetic structure, principal component analysis, and phylogenetic relationships supported each other. That is, the *SBP* genes of *N. incisum* and *N. forrestii* can be clearly distinguished in four species as a large monophyletic branch, respectively. However, the *SBP* genes of *N. oviforme* and *N. franchetii* clustered together. The results support Yang et al. (2019)’s differentiation study of *Notopterygium* species based on multiple genomic fragments and morphological evidence [33]. According to the division of genetic differentiation level among populations, the results of *F*st in this study were consistent with the above results. Therefore, the differentiation of *SBP* gene in *Notopterygium* plants was in accordance with the differentiation level of *Notopterygium* species. The degree of species nucleotide diversity can be influenced by its origin [55]. In our study, the *SBP* gene of *N. oviforme* had the highest nucleotide diversity level, followed by *N. forrestii*, *N. franchetii*, and *N. incisum*, which had the lowest. This study supports the hybridization origin of *N. oviforme* and *N. forrestii* [30].

### 3.4. Expression Pattern and Function Prediction of SBPs

As rooted and fixed organisms, land plants are unable to move and constantly face various unfavorable challenges [56]. In order to adapt to various stimuli, plants have evolved complex signal transduction pathways to sense various stress signals and coordinate their growth [57]. miR156 or miR156-SPL modules play an important role in plant growth and development, as well as in biotic and abiotic stresses [58,59,60]. Genetic analysis of the *SBP* gene family in *Notopterygium* species indicated that all members of the subfamilies I, III, VI, and VIII were regulated by miR156, which induced the expression of downstream genes. Additionally, each member of gene family plays a role in different regulatory pathways; the interaction of multiple members of gene family also performs an indispensable function in some aspects [61]. The results of protein interaction network showed that SBP transcription factors of *Notopterygium* plants mainly interact with AP2-like, AGL, and MYB proteins, which play an important role in regulating flowering and responding to biotic and abiotic stresses [62,63,64].

Based on the RNA-seq data, the expression patterns of *SBP* genes were consistent among the four *Notopterygium* plants; namely, most *SBP* genes were not species-specific. However, these genes had obvious tissue specificity, which suggests that they have different functions or regulatory mechanisms in different tissues or organs. In addition, the expression patterns of the same subfamily genes varied considerably across tissues, suggesting that genes of the same subfamily were more functionally differentiated in different tissues. This result has also been confirmed in other species. For example, regarding *AtSPL10* and *AtSPL11* located in the sixth subfamily, it can be said that *AtSPL10* is essential in fruit pod development; however, *AtSPL11* mainly acts on the flowering signaling pathway in *Arabidopsis thaliana* [65,66].

We detected the expression of seven *NinSBPs* in the living leaves and root tissues of *N. franchetii* under drought and high temperature stress through qRT-PCR. The results showed that under the same stress, the response patterns of the same gene in different tissues were different; in the same tissues, the responses of the same gene under different stress were also different. Therefore, the response patterns of these *SBP* genes are stress-specific and tissue-specific. This result was also confirmed in the enzyme activity and content. In addition, the relative expression levels of some genes significantly increased or decreased after two hours, such as *NinSBP08/10/12*, indicating that these genes may have rapid response ability to help plants resist adverse conditions in the short term. Similar phenomena have also been observed in other species [56,67].

Gene silencing of *TaSPL6* enhanced drought tolerance in wheat and exhibited better growth status [68], a result also validated in tea plants [69]. The expression level of *NinSBP10/12* was significantly inhibited in both leaves and roots under drought stress. Additionally, drought-responsive elements were detected in the promoter regions of these two genes. It is speculated that *NinSBP10/12* can enhance the drought resistance of *Notopterygium* plants. Overexpression of *AtSPL1* or *AtSPL12* enhanced heat tolerance in *Arabidopsis* and tobacco [70]. *NinSBP08* was located in the same branch as *AtSPL1/12*, and the expression level of *NinSBP08* increased about seventy-fold after 12 h of heat stress, while *NinSBP08* was hardly expressed without additional stress. The results demonstrated that high temperature stress could activate the expression of *NinSBP08* in leaves of *Notopterygium* plants and make them survive the adverse environment.

## 4. Materials and Methods

### 4.1. Genome Data Collection and SBP Family Genes Identification

The whole genome sequencing data of the three species of *Notopterygium* were obtained from the research group. Genome assemblies for *N. incisum* (1.48 Gb; N50: 16.57 Mb), *N. francheti* (2.04 Gb; N50: 2.81 Mb), and *N. forrestii* (1.36 Gb; N50: 47.22 Mb) showed high BUSCO completeness of 97.03%, 93.38%, and 98.88%, respectively. The other seven species were downloaded from NCBI (*D. carota*, *V. vinifera*, and *A. thaliana*), CGDB (*C. sativum* and *A. graveolens*), cyVerse platform (*A. sinensis*), and Dryad Digital Repository (*Aralia elata* (Miq.) Seem.), respectively. The Pfam database and Blastp software (version 2.14.0) were used to identify *SBP* family genes (PF03110) with an e-value < 1 × 10^−5^, respectively [71,72]. The above two results were combined and redundant sequences were removed. Then, the NCBI-CDD database was utilized to confirm the conserved domains [73], with only the complete SBP-conserved domain preserved. The *SBP* family genes were renamed according to their distribution on the chromosome, and the chromosome mapping was made by TBtools (version 2.376) [74].

### 4.2. Physicochemical Properties, Subcellular Localization, and Protein Structure

All SBP protein sequences were submitted to ExPASy platform for physicochemical property analysis (https://web.expasy.org/protparam/, accessed on 11 April 2025) [75], Wolf PSORT (https://wolfpsort.hgc.jp/, accessed on 24 April 2025) for subcellular localization, and TMHMM (https://services.healthtech.dtu.dk/services/TMHMM-2.0/, accessed on 6 May 2025) for transmembrane domain prediction [76,77]. The tertiary structure was obtained by homology modeling with SWISS-MODEL (https://swissmodel.expasy.org/, accessed on 27 May 2025) [78].

### 4.3. Multiple Sequence Alignments and Phylogenetic Analysis

Amino acid sequences of the *SBP* family genes from 10 species were used to construct a phylogenetic tree using MEGA X software (version 2.056) [79]. First, multiple alignment was conducted using muscle; then, the phylogenetic tree was constructed using the maximum-likelihood (ML) method with the best amino acid substitution model JTT + G and 1000 bootstrap replicates. Furthermore, the ML tree was submitted to iTol website for phylogenetic tree beautification [80].

### 4.4. Gene Structure and Conserved Motif Analysis

The conserved motifs of SBP proteins were identified by the program MEME with a maximum of 15 motifs and other default settings [81]. The gene structure display server (GSDS) was utilized to investigate the *SBP* gene structures [82]. TBtools software (version 2.376) was used to visualize gene structure and conserved motif composition.

### 4.5. Cis-Element Analysis and miRNA156-Targeting SBPs Prediction

The 2000 bp upstream of the *SBP* family genes was extracted as the promoter sequence and submitted to PlantCARE database for *cis*-element analysis [83]. Although three genes (*NinSBP11*, *NinSBP20*, and *NfrSBP06*) showed partial overlaps with adjacent genes, we maintained the uniform 2000 bp promoter length for all genes to ensure consistency with previous studies and facilitate comparative analyses. The potential targets of miR156 were predicted by psRNATarget server [84].

### 4.6. Protein Interaction Network

In order to explore the interaction of SBP proteins in *Notopterygium* species, NinSBPs were used as representatives to construct a protein interaction network of this genus using the STRING (https://cn.string-db.org/ (accessed on 29 June 2025) [85].

### 4.7. Collinearity and Duplication Type of SBP Genes

The interspecies and intraspecific SBP protein sequences of *Notopterygium* species were compared using Diamond software (version 0.8.22.84) [86]. Then, the MCScanX software (version 2.056) was used to search the collinearity blocks. The duplication types of the *SBP* genes were classified using the duplicate_gene_classifier program, which was incorporated in the MCScanX software (version 2.056) [87]. Finally, *SBP* family genes located in the collinear blocks were extracted using TBtools software (version 2.376).

### 4.8. Gene Duplication/Loss Analysis and Ka/Ks Calculation

First, the OrthoFinder software (version 2.5.4) was adopted to construct a species tree of the 10 species, with default parameters [88]. The species tree was then compared with the gene tree for lineage comparison with Notung software (version 2.1.9.5) for gene replication and loss estimation [89]. To analyze the selection pressure during gene evolution of *NinSBPs*, the synonymous mutation frequency (*Ks*), nonsynonymous mutation frequency (*Ka*), and *Ka/Ks* of collinear gene pairs were calculated employing the Simple *Ka/Ks* Calculator (NG) program in TBtool software (version 2.376)

### 4.9. Genetic Structure and Genetic Diversity of SBP Gene Family

Forty-eight individuals of 16 populations from four *Notopterygium* species were collected and the whole genome was resequenced on BGISEQ-500 platform (Figure S1). The *N. incisum* genome was used as the reference genome for sequence alignment and single-nucleotide polymorphism (SNP) detection, followed by the SNP data of the *SBP* gene family, which was extracted according to the position information of *SBP* family genes on chromosome. SNPs filtering was performed using Vcftools software (version 0.1.17) [90]. Additionally, variant annotation was carried out using snpEff v4.3 software [91].

Based on the SNPs data filtered by linkage disequilibrium (LD: R^2^ < 0.2) with PLINK v1.9 software [92], the genetic structure of the *SBP* gene family was estimated using ADMIXTURE v1.3.0 software and mapping by Pong software (version 1.5) [93,94]. Principal component analysis was performed using PLINK (version 1.90) as well as phylogenetic trees, which were calculated using IQ-TREE software (version 2.1.3) [95]. Nucleotide diversity (*θ*_π_) and genetic differentiation coefficients (*F*st) were obtained using the --windows-pi and --fst windows parameters in Vcftools (version 0.1.17), with the sliding window set to 10 K and the step size set to 5 K.

### 4.10. Expression Pattern Analysis

The expression pattern of *NinSBPs* in root tissues of 45 individuals from 15 populations of four *Notopterygium* species, as well as in different tissues of each species, was studied by RNA-seq (Table S3). These plant materials were collected from Gansu, Qinghai, Sichuan, and Shaanxi provinces. Fresh materials were rapidly frozen with liquid nitrogen and transcriptome sequencing was performed on an Illumina HiSeq X Reagent platform (Majorbio, Shanghai, China). Fastp software (version 23.4) was used for filtering and quality control of the original sequencing data [96]. The gene expression abundance was performed using the comparison software HISAT2 (version 2.10) and the assembly and quantification software StringTie (version 1.3.5 ) [97,98], taking the *N. incisum* genome sequence as reference. The TPM values (transcripts per million reads) were then calculated utilizing the RPKM/FPKM or TPM Calculator plugin in TBtools (version 2.376), and a heat map was drawn by logarithm.

### 4.11. Plant Materials and Seedling Treatments of Abiotic Stress Experiments

The plant material used in this study was 3-year-old cultivated *N. franchetii* seedlings. The roots of the seedlings were cultivated into pots and the stems and leaves were cut to allow re-germination. Six weeks after germination, they were subjected to drought and high-temperature stress treatments. Under drought stress, leaves and roots were collected at 0, 2, 6, 12, and 24 h, after the washed roots were placed in 40% Polyethylene glycol (PEG) 6000 solution. For the heat treatment, the plants were placed in a 40 °C incubator and the leaves and roots were obtained at 0, 2, 6, 12, and 36 h. All samples were collected separately, quickly placed in liquid nitrogen, and stored at −80 °C. Three biological replicates were set for each qRT-PCR experiment.

### 4.12. RNA Extraction and qRT-PCR

Total plant RNA was extracted using the SteadyPure Plant RNA Extraction Kit (Accurate Biotechnology Changsha, China) according to the manufacturer’s instructions. Hifair^®^ III 1st Strand cDNA Synthesis SuperMix for qPCR (gDNA digester plus) was utilized to synthesize the first strand cDNA (Yeasen Biotechnology, Shanghai, China). qRT-PCR experiments were performed with Hieff^®^ qPCR SYBR Green Master Mix (No Rox) Fluorescence Quantification Kit (Yeasen Biotechnology, Shanghai, China). Actin8 was employed as an internal reference gene for normalization and all primer sequences were designed through Primer Premier v6.0 software (Table S4). The relative expression of each gene was calculated according to the 2^−ΔΔCT^ method and significance analysis was performed using Tukey test in Origin software (version10.1.0.78) [99].

### 4.13. Physiological Indexes Determination

The superoxide dismutase (SOD) and peroxidase (POD) activity and malondialdehyde (MDA) content of leaves and roots treated for different times were determined by the SOD, POD, and MDA assay kits (A001-3, A084-3-1 and A003-1) (Nanjing Institute of Biological Engineering, Nanjing, China).

## 5. Conclusions

In this study, 21, 18, and 18 *SBP* family genes were identified in the whole genome of *N. incisum*, *N. francheti*, and *N. forrestii*, respectively, and classified into eight subfamilies. Members of the same subfamily had similar structures and conserved motif compositions, suggesting that they may have similar biological functions. Promoter cis-regulatory element analysis showed that the *SBP* gene may play an important role in the growth and environmental adaptation of *Notopterygium* species. Evolutionary analysis revealed that there was a collinear relationship between the *SBP* gene of *N. incisum* and other closely related Apiaceae species. The expansion of the *SBP* gene family in *Notopterygium* plants was mainly caused by WGD/segmental duplication and transposable element duplication. The *SBP* gene family has experienced severe contraction events, and most of the gene copies have undergone purification selection during evolution. A few genes have recently differentiated, which may lead to the differentiation of new functions and promote the adaptation of *Notopterygium* species to the changing environment. Moreover, there was a clear interspecific genetic differentiation pattern in the *SBP* gene of *Notopterygium* plants, which was consistent with the genetic structure at the species level. Genetic regulation analysis indicated that a total of twenty-nine *SBP* genes were regulated by miR156. The expression patterns of *SBP* genes in *Notopterygium* species were not species-specific, but they were obviously tissue-specific. The results of qRT-PCR experiments illustrated that most of the validated *SBP* genes were responsive to drought and high temperature stresses, but the response patterns were inconsistent. Furthermore, *NinSBP08* and *NinSBP10/12* may have important effects on heat tolerance and drought tolerance, respectively.

## Figures and Tables

**Table 1:** Characteristics of *SBP* family genes in the three *Notopterygium* species.

Name	Gene ID	CDS (bp)	Exon	Protein			Subcellular Localization
				Aa	Mw (kDa)	Ip	
*NinSBP01*	evm.model.Chr01.784	2931	11	976	108,949.93	7.71	Nucleus
*NinSBP02*	evm.model.Chr02.2860	960	3	319	35,805.74	8.46	Nucleus
*NinSBP03*	evm.model.Chr02.2870	1377	4	458	50,302.85	7.79	Nucleus
*NinSBP04*	evm.model.Chr04.2978	3267	10	1088	119,719.87	7.75	Nucleus/plasma membrane
*NinSBP05*	evm.model.Chr04.4291	1620	3	539	59,630.86	6.45	Nucleus
*NinSBP06*	evm.model.Chr04.4446	1101	3	366	40,947	8.88	Nucleus
*NinSBP07*	evm.model.Chr05.1247	1839	7	612	69,337.78	9.18	Nucleus
*NinSBP08*	evm.model.Chr05.1249	1014	3	337	38,098.89	9.25	Nucleus
*NinSBP09*	evm.model.Chr05.1491	1611	3	536	59,371.72	7.64	Nucleus
*NinSBP10*	evm.model.Chr06.2957	1116	3	371	40,026.02	9.01	Nucleus
*NinSBP11*	evm.model.Chr08.3100	1125	3	374	41,660.23	7.66	Nucleus
*NinSBP12*	evm.model.Chr08.3801	411	2	136	15,871.68	8.77	Nucleus
*NinSBP13*	evm.model.Chr09.2640	540	2	179	20,327.99	9.65	Nucleus
*NinSBP14*	evm.model.Chr09.3014	507	2	168	18,581.51	9.27	Nucleus
*NinSBP15*	evm.model.Chr09.3839	1026	3	341	36,294.7	8.52	Nucleus
*NinSBP16*	evm.model.Chr10.473	1173	3	390	43,878.52	8.03	Nucleus
*NinSBP17*	evm.model.Chr10.476	918	3	305	34,890.28	8.68	Nucleus
*NinSBP18*	evm.model.Chr11.935	2403	10	800	89,469.54	6	Nucleus
*NinSBP19*	evm.model.Chr11.3220	897	3	298	34,161.53	8.87	Nucleus
*NinSBP20*	evm.model.Chr11.3843	426	2	141	16,213.3	9.01	Nucleus
*NinSBP21*	evm.model.Chr11.4037	414	2	137	16,025.04	8.86	Nucleus
*NfrSBP01*	evm.model.Chr04.2435	2931	11	976	108,740.62	7.05	Nucleus
*NfrSBP02*	evm.model.Chr09.3138	2991	11	996	110,688.17	6.58	Nucleus
*NfrSBP03*	evm.model.Chr09.4323	1377	4	458	50,304.82	7.79	Nucleus
*NfrSBP04*	evm.model.Chr09.4337	1110	6	369	41,115.95	8.42	Nucleus
*NfrSBP05*	evm.model.Chr09.4286	966	3	321	35,882.82	8.74	Nucleus
*NfrSBP06*	evm.model.Chr02.5311	3288	10	1095	120,367.73	7.73	Nucleus/plasma membrane
*NfrSBP07*	evm.model.Chr02.3783	1623	3	540	59,818.25	7.01	Nucleus
*NfrSBP08*	evm.model.Chr02.3760	1623	3	540	59,887.27	6.83	Nucleus
*NfrSBP09*	evm.model.Chr02.3588	1101	3	366	40,916.89	8.79	Nucleus
*NfrSBP10*	evm.model.Chr08.112	1143	4	380	42,584.04	8.83	Nucleus
*NfrSBP11*	evm.model.Chr08.533	1611	3	536	59,414.7	7.01	Nucleus
*NfrSBP12*	evm.model.Chr03.4221	411	2	136	15,885.7	8.77	Nucleus
*NfrSBP13*	evm.model.Chr01.7697	537	2	178	20,356.04	9.7	Nucleus
*NfrSBP14*	evm.model.Chr01.10377	1065	3	354	37,817.48	8.52	Nucleus
*NfrSBP15*	evm.model.Chr01.2531	2388	10	795	88,797.73	5.74	Nucleus
*NfrSBP16*	evm.model.Contig1761.2	507	2	168	18,567.48	9.27	Nucleus
*NfrSBP17*	evm.model.Contig2298.2	918	3	305	34,772.06	8.69	Nucleus
*NfrSBP18*	evm.model.Contig711.2	1116	3	371	40,197.36	8.82	Nucleus
*NfoSBP01*	evm.model.tig75.187	3126	16	1041	115,888.98	6.78	Nucleus
*NfoSBP02*	evm.model.tig30.821	2991	11	996	110,808.21	6.48	Nucleus
*NfoSBP03*	evm.model.tig30.1437	1467	6	488	53,896.91	7.05	Nucleus
*NfoSBP04*	evm.model.tig30.1454	1116	5	371	41,606.75	8.77	Nucleus
*NfoSBP05*	evm.model.tig13.592	3267	10	1088	119,930.04	7.74	Nucleus/plasma membrane
*NfoSBP06*	evm.model.tig13.1554	1623	3	540	59,935.29	6.53	Nucleus
*NfoSBP07*	evm.model.tig13.1668	1101	3	366	40,974.97	8.69	Nucleus
*NfoSBP08*	evm.model.tig50.933	1611	3	536	59,326.7	7.62	Nucleus
*NfoSBP09*	evm.model.tig10.1133	1125	3	374	41,658.26	7.66	Nucleus
*NfoSBP10*	evm.model.tig10.1754	417	4	138	15,896.9	7.06	Nucleus
*NfoSBP11*	evm.model.tig14.564	3225	10	1074	119,249.91	8.35	Nucleus/plasma membrane
*NfoSBP12*	evm.model.tig14.437	507	2	168	18,581.51	9.27	Nucleus
*NfoSBP13*	evm.model.tig24.40	1026	3	341	36,266.61	8.34	Nucleus
*NfoSBP14*	evm.model.tig33.125	1185	3	394	44,194.87	8.03	Nucleus
*NfoSBP15*	evm.model.tig33.124	918	3	305	34,811.18	8.69	Nucleus
*NfoSBP16*	evm.model.tig25.331	1830	9	609	67,913.96	5.9	Nucleus
*NfoSBP17*	evm.model.tig25.1991	897	3	298	34,201.55	8.51	Nucleus
*NfoSBP18*	evm.model.tig40.43	414	2	137	16,016.02	8.86	Nucleus

**Table 2:** Prediction of miR156 regulatory loci of *SBP* family genes in three *Notopterygium* species.

	*NinSBPs*	*NfrSBPs*	*NfoSBPs*
I	*NinSBP02*, *NinSBP06*, *NinSBP07*, *NinSBP08*, *NinSBP11*	*NfrSBP04*, *NfrSBP05*, *NfrSBP09*, *NfrSBP10*	*NfoSBP04*, *NfoSBP07*, *NfoSBP09*
III	*NinSBP05*, *NinSBP09*	*NfrSBP07*, *NfrSBP08*, *NfrSBP11*	*NfoSBP06*, *NfoSBP08*
VI	*NinSBP03*, *NinSBP16*	*NfrSBP03*	*NfoSBP03*, *NfoSBP14*
VIII	*NinSBP10*, *NinSBP15*	*NfrSBP14*, *NfrSBP18*	*NfoSBP13*

**Table 3:** The distribution of SNPs within different regions.

Number of Effects by Region	Count	Percent
DOWNSTREAM	209	28.09%
EXON	300	40.32%
INTRON	195	26.21%
SPLICE_SITE_REGION	7	0.94%
UPSTREAM	10	1.34%
UTR_3_PRIME	16	2.15%
UTR_5_PRIME	7	0.94%

**Table 4:** Chromosome distribution of SNPs.

Chromosome	Length	Variants
1	157,866,847	27
2	153,106,864	21
4	129,796,441	288
5	129,440,675	36
6	125,876,602	2
8	116,547,418	5
9	101,813,303	27
10	94,455,821	22
11	93,597,197	97
Total	1,102,501,168	525

## Data Availability

The original contributions presented in this study are included in the article/Supplementary Material. Further inquiries can be directed to the corresponding authors.

## Supplementary Materials

The following supporting information can be downloaded at https://www.mdpi.com/article/10.3390/ijms27020979/s1.
