# Supplementary material for: Evolutionary History, Transcriptome Expression Profiles, and Abiotic Stress Responses of the SBP Family Genes in the Three Endangered Medicinal Notopterygium Species
Source: Int J Mol Sci. 2026 Jan 19;27(2):979. doi: 10.3390/ijms27020979 (PMC12841832; doi:10.3390/ijms27020979)
Supplement: Supplementary file 1 [file ijms-27-00979-s001.zip › Supplemental Figures.pdf]

Appendix

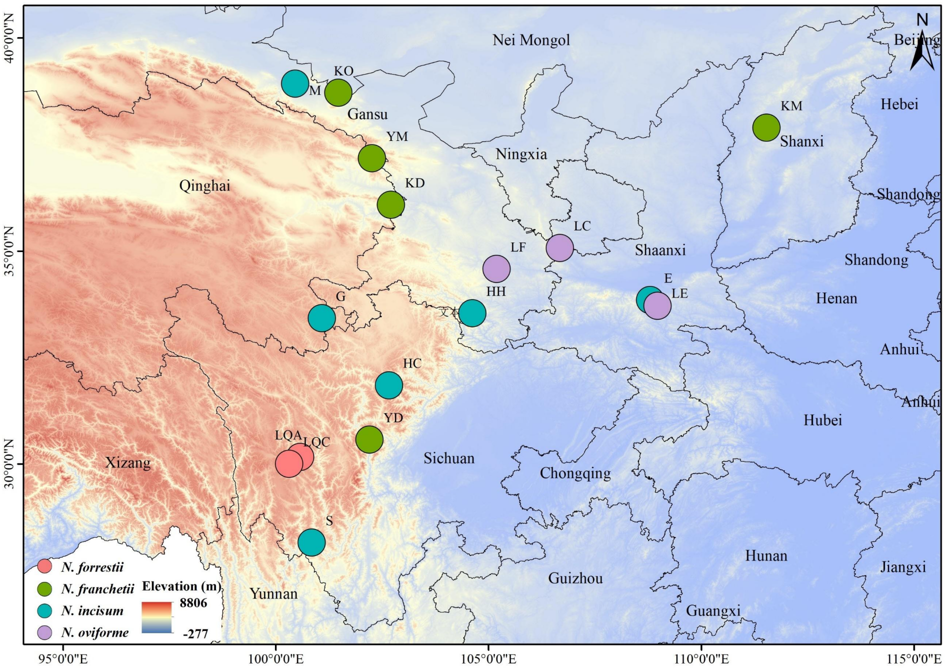

Figure S1. The geographical distribution of sampled populations of the four *Notopterygium* species.

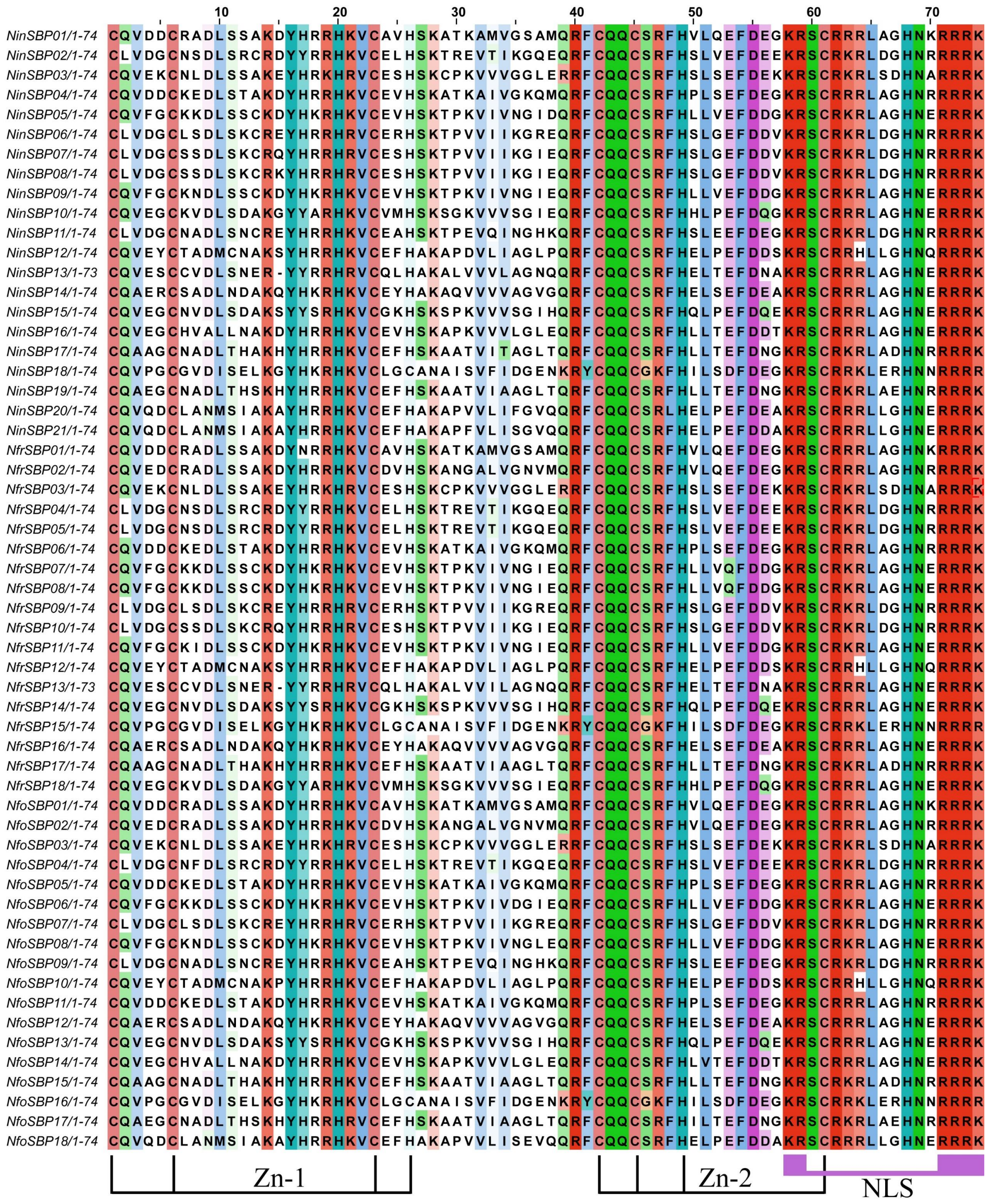

Figure S2. Multiple alignment of conserved domains of SBP family amino acid sequences in *N. incisum*, *N. franchetii* and *N. forrestii*.

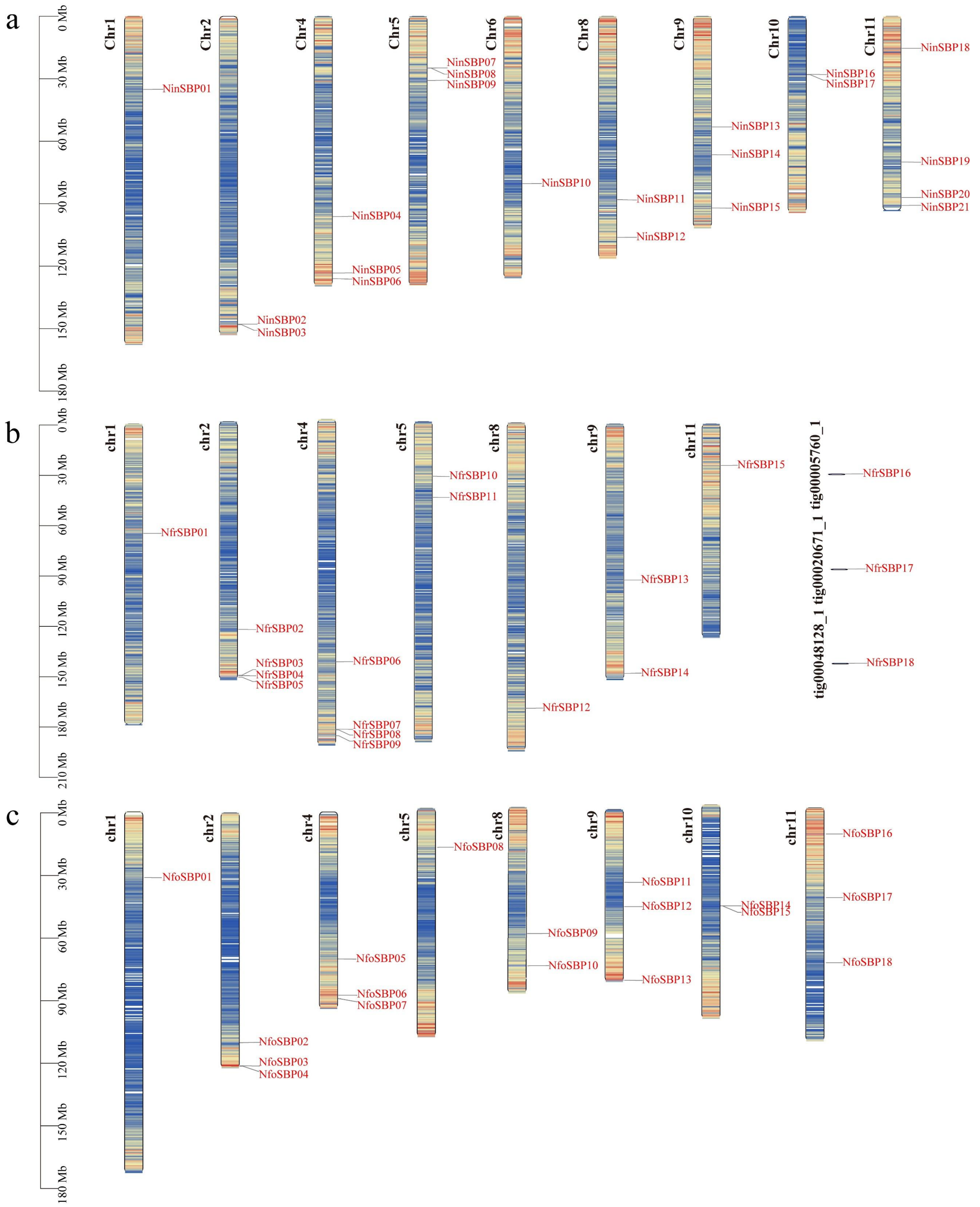

Figure S3. Distribution of *SBP* family genes on each chromosome in the three *Notopterygium* species. (a) *N. incisum*; (b) *N. franchetii*; (c) *N. forrestii*.

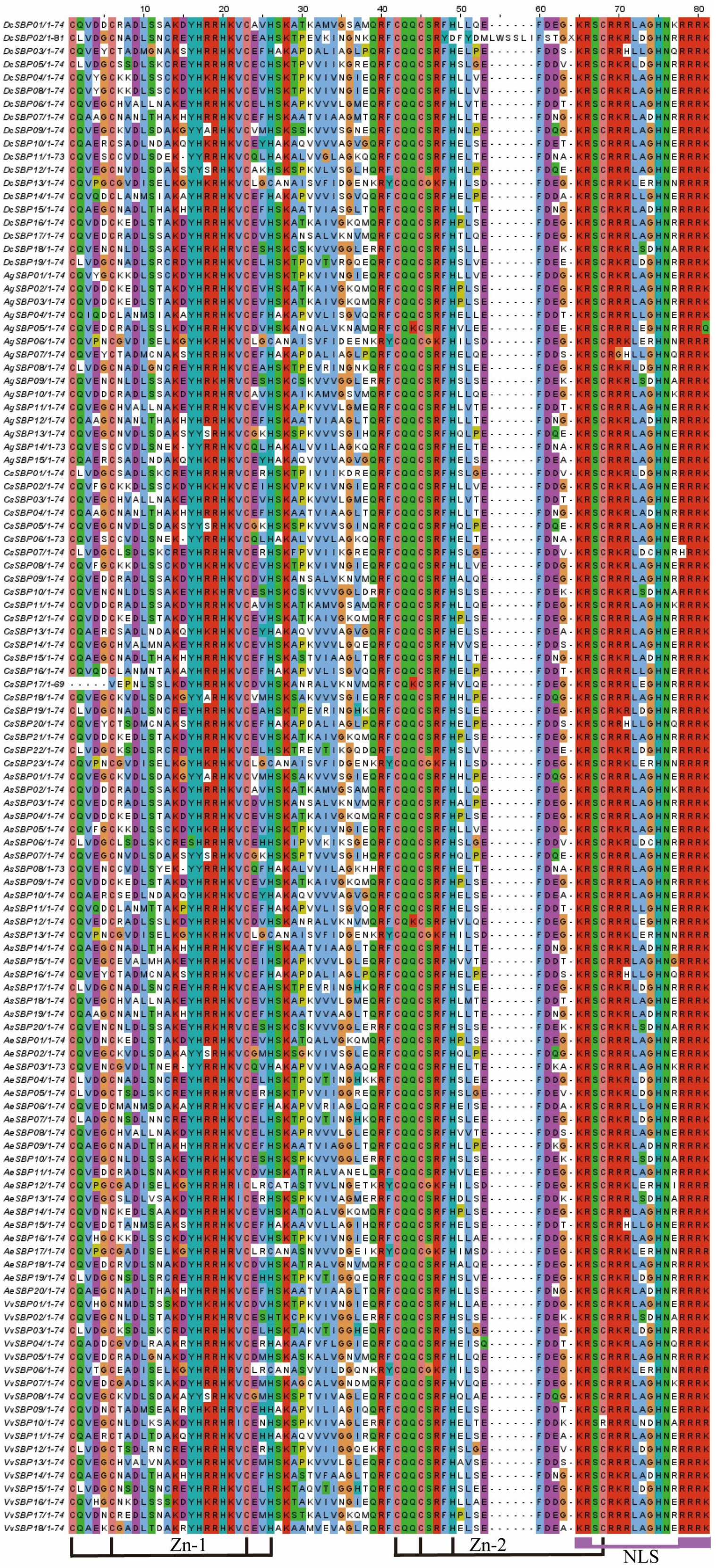

Figure S4. Multiple alignment of conserved domains of SBP family amino acid sequences in the other six species, include carrot, celery, coriander, *A. sinensis, A. elata* and grape.

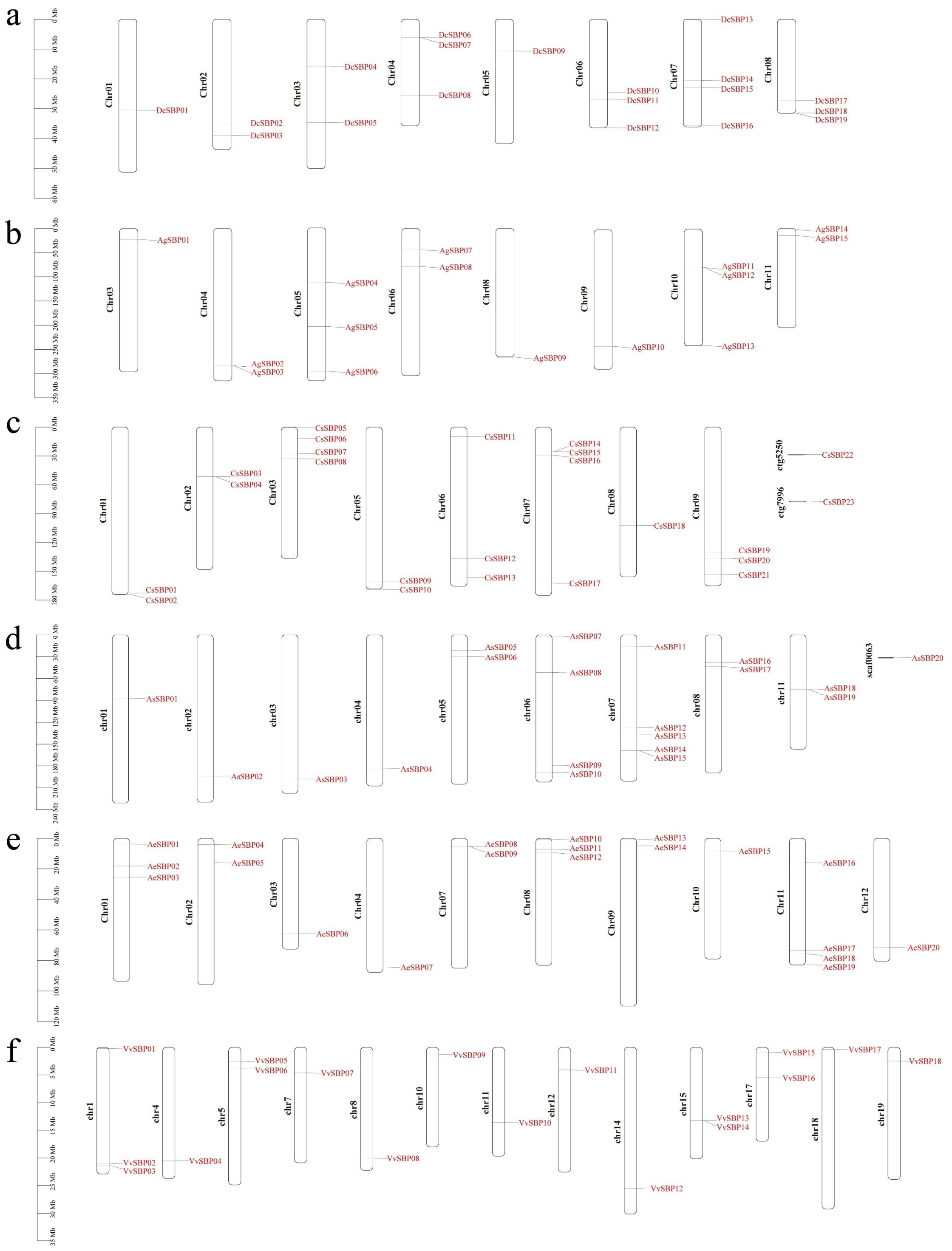

Figure S5. Distribution of *SBP* family genes on each chromosome in the other six species. (a) carrot; (b) celery; (c) coriander; (d*) A. sinensis;* (e) *A. elata;* (f) grape.

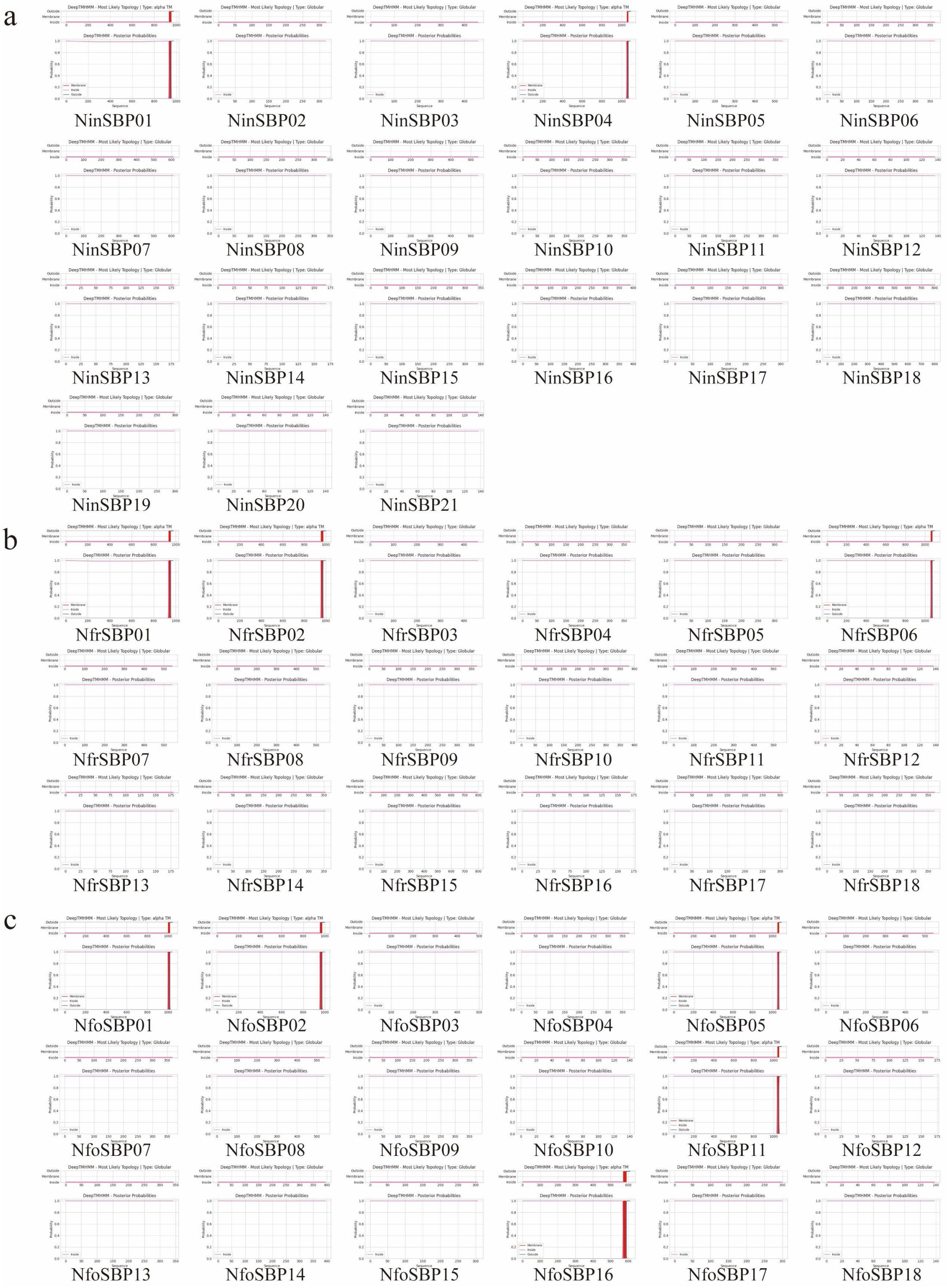

Figure S6. Prediction of transmembrane structure of SBPs proteins in the three *Notopterygium* species. (a) *N. incisum*; (b) *N. franchetii*; (c) *N. forrestii*.

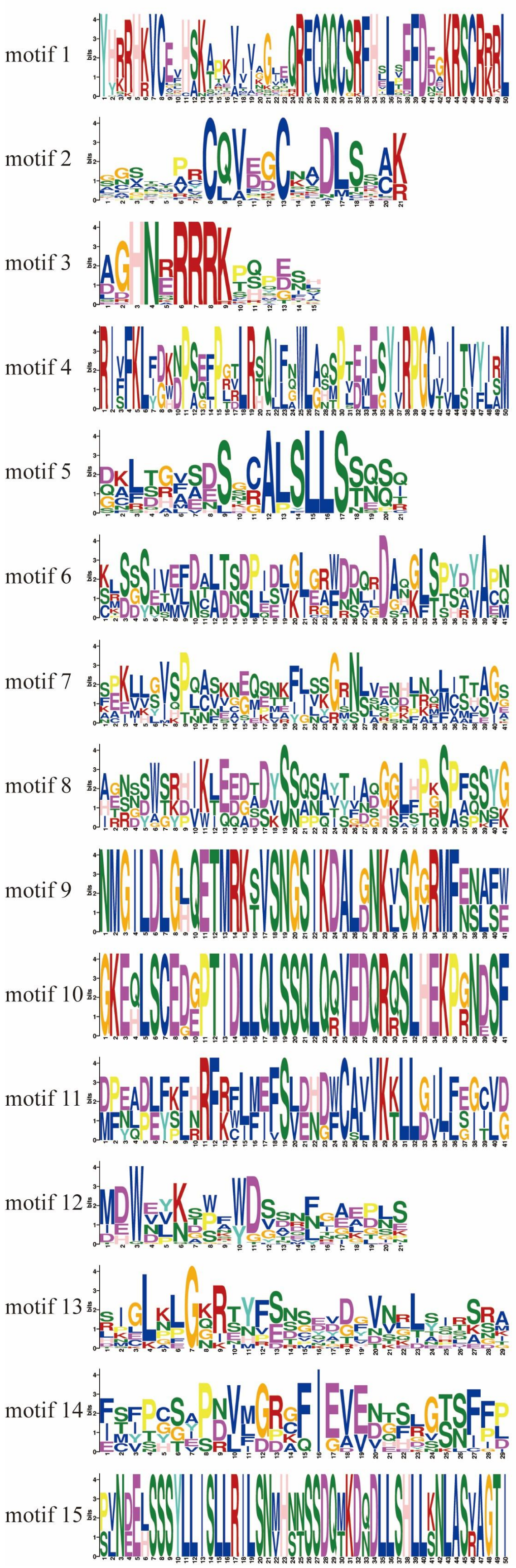

Figure S7. Motif structures of SBP family genes in the three *Notopterygium* species.

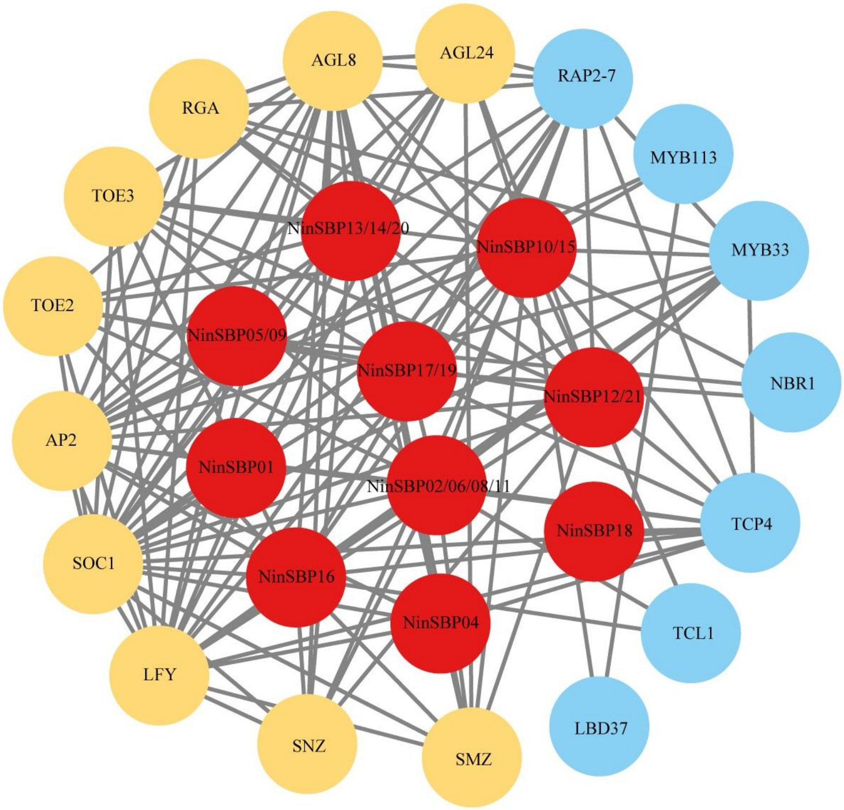

Figure S8. Protein interaction network of NinSBPs family genes. Red Circle represents NinSBPs proteins, yellow circle represents flowering relate proteins, and blue circle represents stress resistance related proteins.

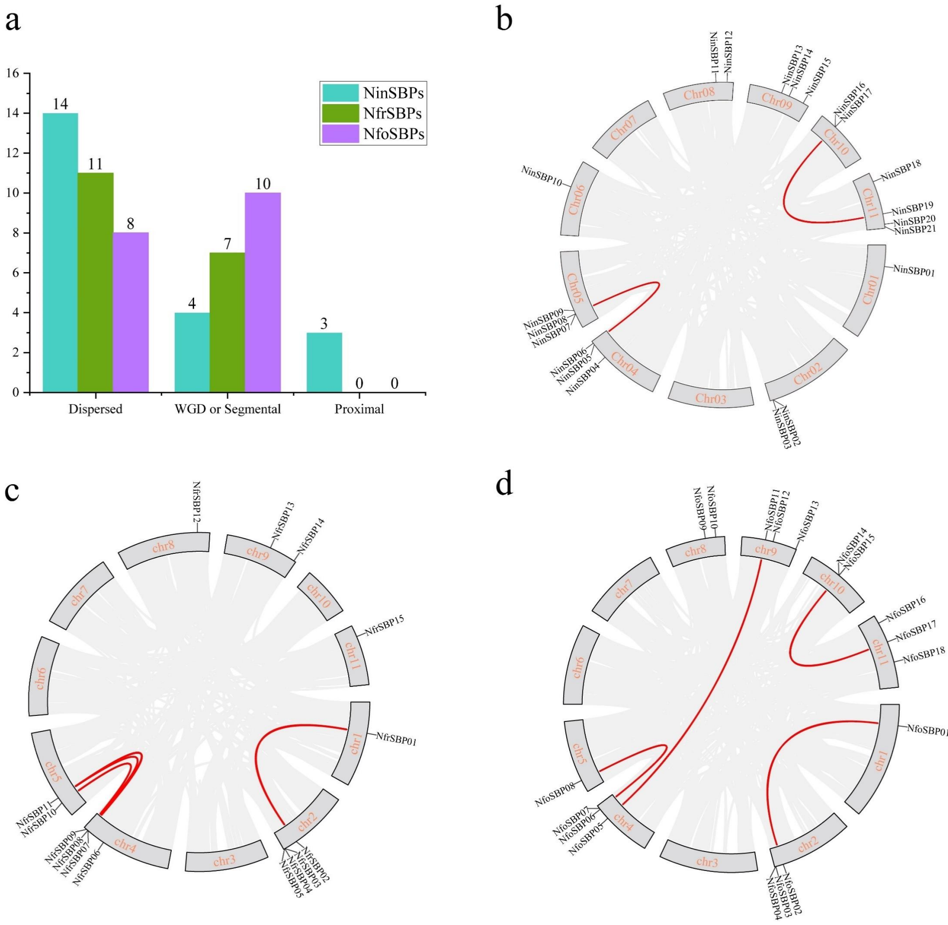

Figure S9. The patterns of *SBP* gene replication and collinearity analysis of *NinSBPs*, *NfrSBPs* and *NfoSBPs*, respectively. (a) Gene replication type; (b) *NinSBPs* collinearity gene pairs; (c) *NfrSBPs* collinearity gene pairs; (d) *NfoSBPs* collinearity gene pairs.

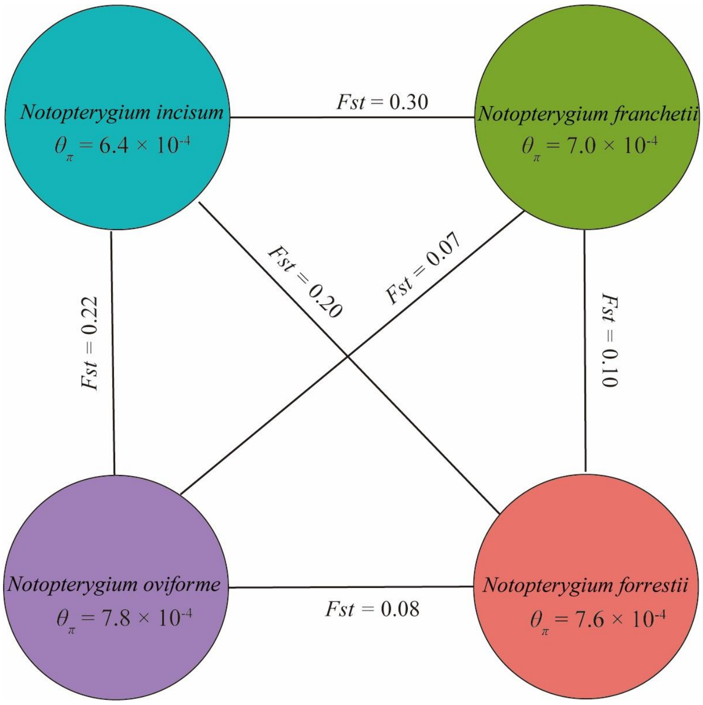

Figure S10. Genetic differentiation index (*F*st) and Nucleotide diversity (π) of *Notopterygium* species.

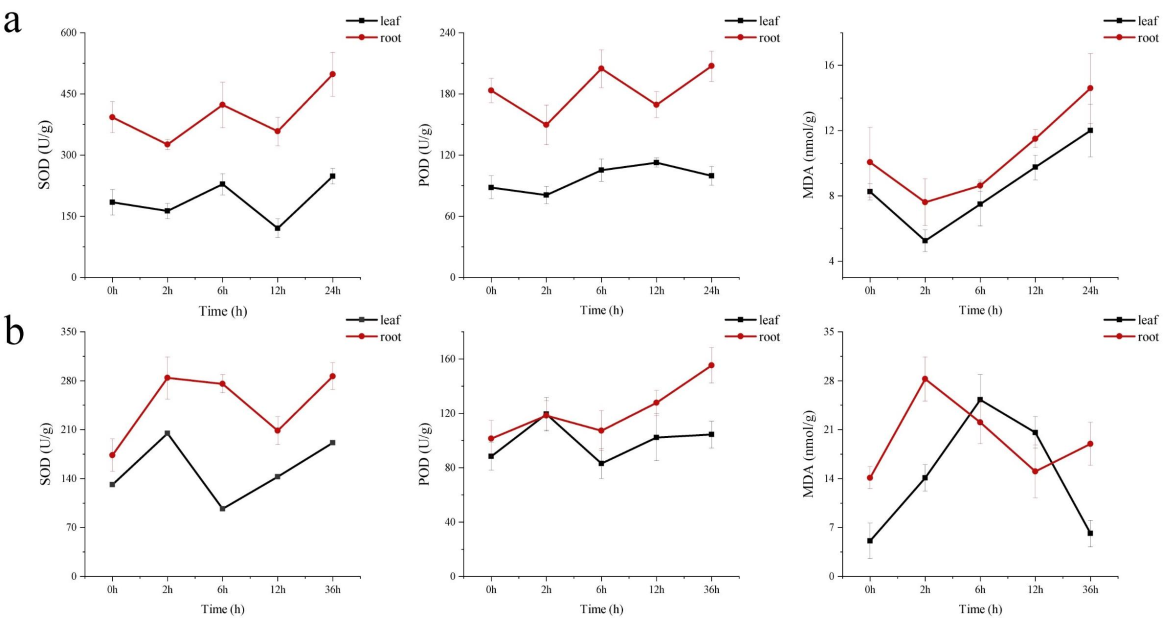

Figure S11. Physiological index determination of leaves and roots in *N. franchetii* under drought and high-temperature stresses. (a) Drought stress; (b) High-temperature stress.
